# Severe skeletal and renal complications in SLC4A1-related distal renal tubular acidosis following prolonged treatment interruption: a case report highlighting transition-related vulnerability

**DOI:** 10.1186/s12882-026-05213-4

**Published:** 2026-08-08

**Authors:** Xinying Gao, Nan Bai, Xuejing Sun, Yao Yao

**Affiliations:** 1 Department of Pediatrics, Beijing Geriatric Hospital, Beijing, China; 2 Department of Pediatrics, Beijing Liang Xiang Hospital, Beijing, China; 3 Department of Respiratory Medicine, Beijing Children’s Hospital, Capital Medical University, National Center for Children’s Health, No. 56 Nanlishi Road, Xicheng District, Beijing, 100045 China

**Keywords:** Distal renal tubular acidosis, Nephrocalcinosis, Nephrolithiasis, Osteomalacia, Skeletal complications

## Abstract

**Background:**

SLC4A1-related distal renal tubular acidosis (dRTA), caused by dysfunction of the anion exchanger 1 (AE1), is typically associated with a relatively mild and late-onset phenotype. However, outcomes may vary substantially, particularly in the setting of delayed diagnosis or prolonged interruption of therapy.

**Case presentation:**

We report a 31-year-old woman with genetically confirmed SLC4A1-related dRTA who presented with recurrent nephrolithiasis, urinary tract infections, and severe skeletal deformities. She discontinued alkali therapy for approximately 13 years after childhood diagnosis, resulting in extreme growth retardation (height −6.2 SD) and advanced osteomalacia. Laboratory evaluation revealed severe hyperchloremic metabolic acidosis, hypokalemia, persistently alkaline urine, and mildly reduced renal function consistent with chronic kidney disease (CKD) stage 2. Imaging demonstrated Looser–Milkman pseudofractures, “fish vertebrae,” and nephrocalcinosis with ureteral obstruction requiring double-J stent placement. Genetic testing identified a heterozygous SLC4A1 variant (c.2704G>T, p. Asp902Tyr), classified as likely pathogenic. The patient’s mother, carrying the same variant, exhibited a milder phenotype.

**Conclusions:**

This case illustrates that prolonged interruption of alkali therapy in SLC4A1-related dRTA may result in severe and irreversible skeletal and renal complications, even in genotypes typically considered mild. It highlights the pediatric-to-adult transition as a critical period for treatment discontinuation and underscores the importance of lifelong therapy, early intervention, and long-term follow-up.

**Supplementary Information:**

The online version contains supplementary material available at https://doi.org/10.1186/s12882-026-05213-4.

## Introduction

Hereditary distal renal tubular acidosis (dRTA), also known as type 1 renal tubular acidosis, is a rare inherited disorder characterized by hyperchloremic metabolic acidosis and impaired urinary acidification due to dysfunction of α-intercalated cells in the collecting duct [1–3].

The estimated global prevalence is approximately 1 in 100,000 live births. Hereditary forms are primarily caused by mutations in *SLC4A1*, *ATP6V1B1*, and *ATP6V0A4* [4]. Clinical manifestations range from asymptomatic disease to growth retardation, rickets or osteomalacia, nephrolithiasis, nephrocalcinosis, and progressive chronic kidney disease (CKD) [5, 6].

*SLC4A1*-related dRTA is generally considered a milder and later-onset form compared with autosomal recessive subtypes; however, substantial phenotypic variability exists, even within affected families. Although early diagnosis and lifelong alkali therapy can prevent most complications [7], treatment adherence is frequently suboptimal during adolescence and early adulthood. The long-term consequences of therapy interruption during this transition period remain insufficiently characterized.

Here, we report a genetically confirmed case of SLC4A1-related dRTA with an unusually severe phenotype following prolonged treatment interruption, highlighting the risk of irreversible complications and the importance of sustained management during the transition to adult care.

## Case presentation

The proband was a 31-year-old woman who presented to the nephrology department with recurrent nephrolithiasis requiring lithotripsy and placement of a ureteral double-J (JJ) stent due to urinary obstruction.

Her medical history was notable for growth retardation identified in childhood, when dRTA was diagnosed at a pediatric center. Intermittent alkali therapy with potassium citrate, prepared as an institutional formulation at that center, was initiated. However, after transition to adulthood, she could no longer continue routine follow-up at the pediatric center, and access to the same citrate preparation became limited. Together with insufficient understanding of the long-term consequences of dRTA, these barriers contributed to discontinuation of alkali therapy for approximately 13 years during adolescence and early adulthood.

During this untreated period, she developed progressive short stature, recurrent urinary tract infections, and recurrent nephrolithiasis, resulting in annual hospitalizations over the past several years.

Family history revealed that the patient’s mother had short stature (150 cm, -2 SD), genu valgum, and was diagnosed with dRTA in adulthood. Arterial blood gas analysis showed hyperchloremic metabolic acidosis (pH 7.37, HCO3- 18.4 mmol/L), hypokalemia, preserved renal function, and alkaline urine (pH 7.5). She had mild dRTA with normal renal function, mild acidosis, and no severe symptoms, and had not received regular alkali therapy. The father and the patient’s younger brother were clinically asymptomatic, with no known renal or skeletal involvement. Available biochemical and blood gas evaluations did not show evidence of metabolic acidosis. Genetic testing was not performed because they had no overt clinical manifestations and declined further testing.

On admission, physical examination revealed severe short stature (120 cm, − 6.2 SD), frontal bossing, marked genu valgum, and multiple joint deformities, consistent with advanced metabolic bone disease.

Laboratory investigations showed severe hyperchloremic metabolic acidosis (pH 7.19, HCO₃⁻ 12.7 mmol/L), hypokalemia, hypophosphatemia, and mild hypocalcemia, together with secondary hyperaldosteronism. Bone metabolism markers demonstrated elevated alkaline phosphatase and a moderately elevated PTH level (228 pg/mL; reference range, 11–67 pg/mL), consistent with secondary hyperparathyroidism. Urinalysis revealed persistently alkaline urine; the available spot urine calcium-to-creatinine ratio was 0.1. Renal function was mildly reduced, corresponding to CKD stage 2 (eGFR 88 mL/min/1.73 m²) (Table 1).

**Table 1:** Key laboratory findings at admission and follow-up

Examination item	Reference range	Admission	6-month follow-up	12-month follow-up / rehospitalization
**Serum biochemicals**				
Potassium (mmol/L)	3.50–5.50	3.00	3.70	3.50
Sodium (mmol/L)	135–145	134	141	140
Chloride (mmol/L)	96.0–108.0	110	106	113
Calcium (mmol/L)	2.11–2.52	2.15	2.20	2.25
Phosphate (mmol/L)	0.85–1.51	0.75	0.80	0.7
Magnesium (mmol/L)	0.73–1.04	0.88	0.8	0.96
Bicarbonate / CO₂ (mmol/L)	22–29	12.7	20	18
ALP (U/L)	15–112	261	148	132
Creatinine (µmol/L)	33.0–75.0	79	NA	74
BUN / urea (mmol/L)	2.1–7.8	7.1	NA	5.17
PTH (pg/mL)	11–67	228	NA	NA
eGFR (mL/min/1.73 m²)	—	88	NA	87
**Arterial blood gas analysis**				
pH	7.35–7.45	7.19	7.35	NA
PO₂ (mmHg)	83.0–108.0	93	95	NA
PCO₂ (mmHg)	35.0–45.0	28	20	NA
Base excess (mmol/L)	-3.0 to 3.0	-10.3	-5	NA
TCO₂ (mmol/L)	24.0–32.0	13	19	NA
**Urine tests**				
Urine specific gravity	1.003–1.030	1.011	NA	1.015
Urine pH	4.5–8.0	7.0	NA	7.5
Calcium-to-creatinine ratio	0–0.2	0.1	NA	NA
Urine occult blood	Negative	Negative	NA	2+ (80 cells/µL)
Urine protein	Negative	1+	NA	2+ (1.0 g/L)
Leukocyte esterase	Negative	1+	NA	3+ (500 cells/µL)
Nitrite	Negative	Negative	NA	Negative
**RASS system**				
Plasma renin (ng/dL)	0.20–3.2	5.4	NA	NA
Aldosterone (ng/dL)	4–31	> 100	NA	NA
ACE2 (ng/dL)	2.5–13	7.9	NA	NA

Abbreviations: ALP, alkaline phosphatase; BUN, blood urea nitrogen; eGFR, estimated glomerular filtration rate; PTH, parathyroid hormone; RASS, renin–angiotensin–aldosterone system; ACE2, angiotensin-converting enzyme 2; TCO₂, total carbon dioxide

NA indicates that the value was not available from the medical records

The 12-month urine findings were obtained during rehospitalization for recurrent urinary tract infection and nephrolithiasis and should be interpreted in that clinical context

Serial PTH, 25-hydroxyvitamin D, urinary phosphate handling, and urinary citrate measurements were not available

**Fig. 1:**
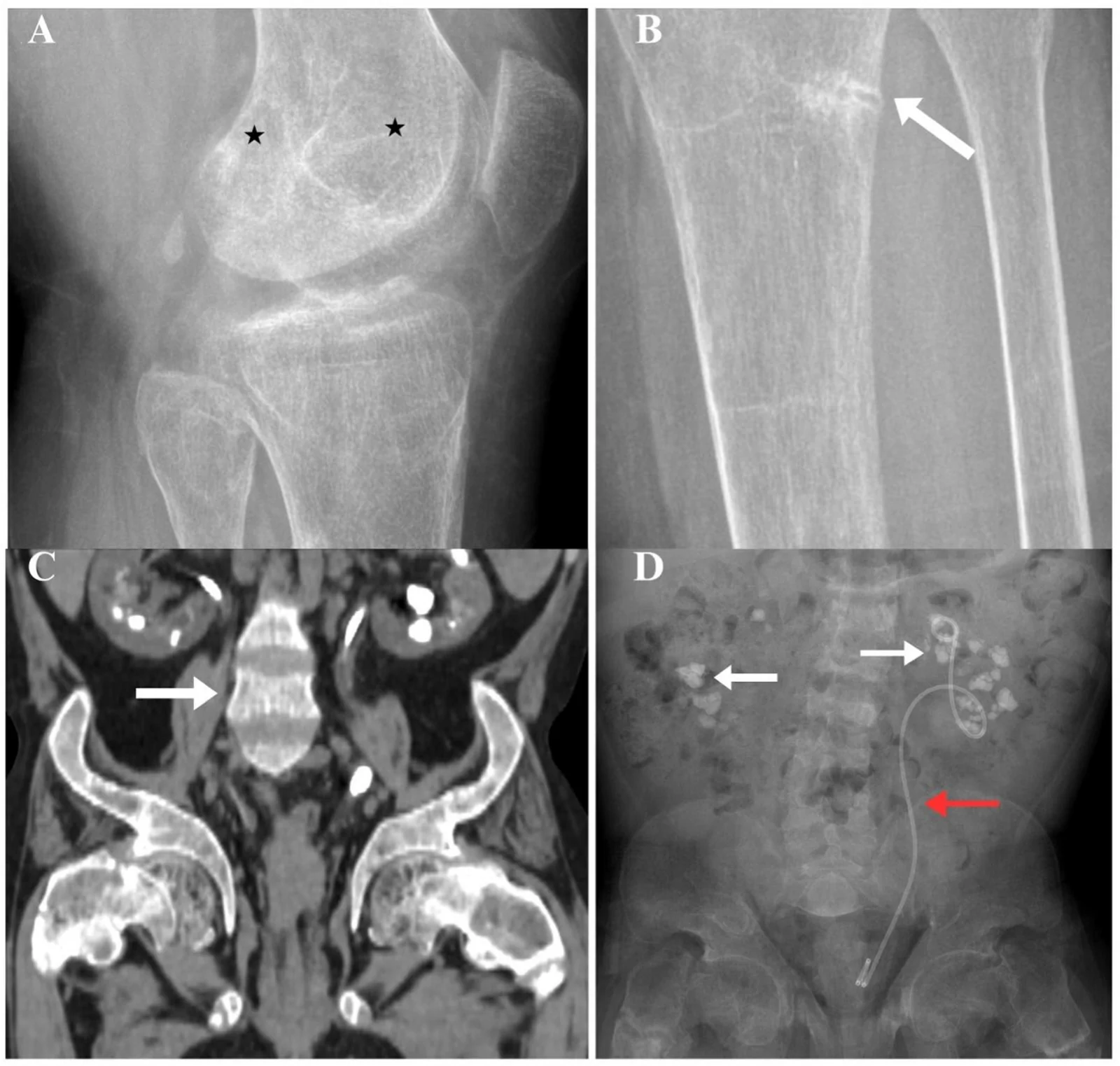
Radiographic features of osteomalacia and nephrocalcinosis. (**A**) Honeycomb appearance (stars). (**B**) Looser-Milkman lines (white arrow). (**C**) Fish vertebrae-like changes (white arrow) and Trefoil Pelvis. (**D**) Extensive renal medullary calcification (white arrow); JJ stent (red arrow)

Radiographs demonstrated severe osteomalacia with characteristic features: Fig. 1A shows generalized osteopenia with a honeycomb trabecular pattern in the distal femoral metaphysis (stars). Figure 1B reveals pathognomonic Looser-Milkman pseudo fractures (white arrows), appearing as linear radiolucent bands perpendicular to the cortex. Figure 1C demonstrates biconcave “fish vertebra” deformities and trefoil pelvis configuration with acetabular protrusion. Figure 1D shows extensive bilateral medullary nephrocalcinosis on computed tomography, with the indwelling ureteral JJ stent visible.

**Fig. 2:**
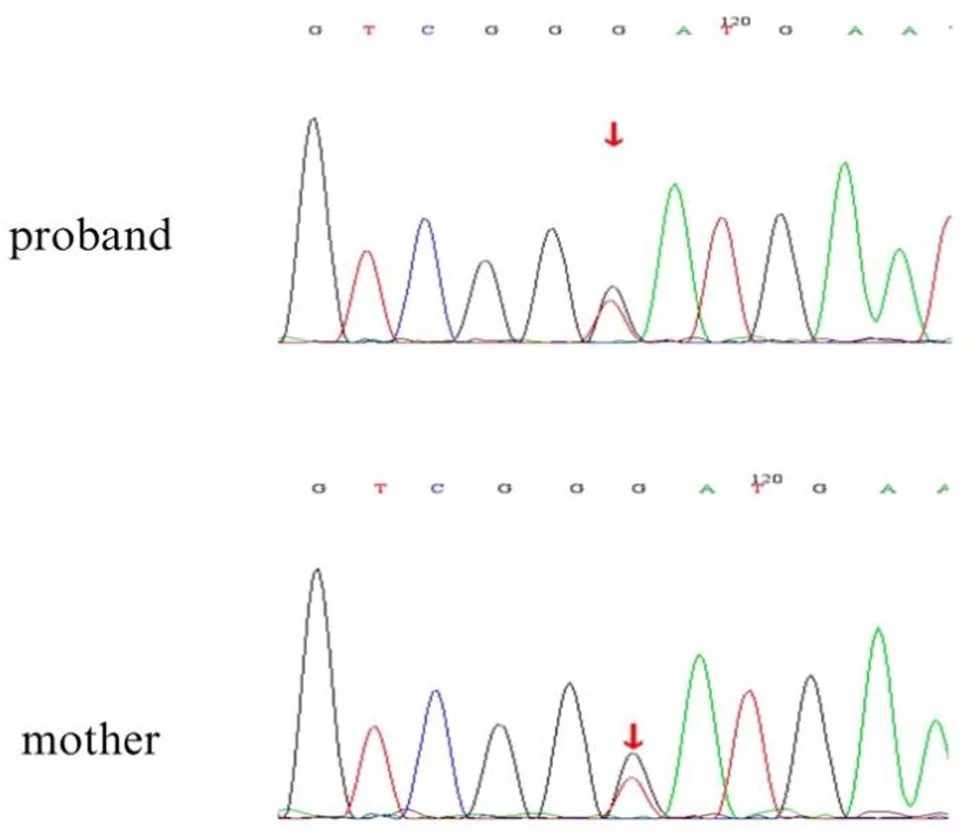
Electropherograms reveal a heterozygous missense variant, c.2704G > T (p. Asp902Tyr), present in both individuals. This variant is classified as likely pathogenic according to the ACMG guidelines (Evidence: PM5_Strong, PM2_Supporting, PP4, PP1)

Genetic analysis revealed a heterozygous missense variant in the *SLC4A1* gene (c.2704G > T, p. Asp902Tyr) (Fig. 2). Co-segregation analysis confirmed the presence of the identical variant in the patient’s mother. In accordance with ACMG guidelines, this variant is classified as likely pathogenic (PM5_Strong + PM2_Supporting + PP4 + PP1), supporting a diagnosis of autosomal dominant dRTA.

Following discharge, potassium citrate therapy with potassium was reinitiated, targeting a serum bicarbonate level > 22 mmol/L [7]. The administered dose (0.79 mmol/kg/day) was below the recommended therapeutic range for active disease (1–3 mmol/kg/day), and potassium supplementation (1.45 mmol/kg/day) was added. Baseline 25-hydroxyvitamin D was unavailable, and calcium or vitamin D supplementation was not started at discharge. No orthopedic intervention was performed. The elevated PTH was interpreted as secondary to chronic metabolic acidosis. No parathyroid-specific treatment was initiated; management focused on correction of acidosis, potassium supplementation, and follow-up of calcium-phosphate and bone metabolic parameters.

At 6-month follow-up, partial metabolic correction was achieved, with pH increasing to 7.35, serum bicarbonate increasing to 20 mmol/L, serum potassium improving to 3.7 mmol/L, and alkaline phosphatase decreasing to 148 U/L. However, serum phosphate remained low at 0.80 mmol/L, and the skeletal deformities remained unchanged. Because post-discharge follow-up was mainly urology-centered, serial monitoring of PTH, 25-hydroxyvitamin D, urinary phosphate handling, urinary citrate, and other mineral metabolic parameters was incomplete.

At 12-month follow-up, the patient was rehospitalized for recurrent urinary tract infection and nephrolithiasis, indicating persistent susceptibility to urological complications despite metabolic improvement. Renal function remained stable (eGFR 87 mL/min/1.73 m²). She remains under long-term follow-up.

## Discussion

dRTA is characterized by impaired acid secretion in the distal nephron, resulting in normal anion gap hyperchloremic metabolic acidosis. Among its genetic causes, mutations in SLC4A1, encoding the anion exchanger 1 (AE1), are a well-recognized cause of autosomal dominant dRTA (AD-dRTA). AE1 is expressed in the basolateral membrane of α-intercalated cells in the collecting duct, where it mediates electroneutral Cl⁻/HCO₃⁻ exchange and plays a critical role in systemic acid–base homeostasis [8]. Compared with autosomal recessive forms, AD-dRTA is typically associated with a milder clinical phenotype and may present as incomplete dRTA in some individuals [9].

A heterozygous SLC4A1 variant (c.2704G > T, p. Asp902Tyr) was identified. This variant has been detected in cohort-based studies of dRTA but has not been extensively characterized [10]. It is located in the C-terminal domain of AE1, a known mutational hotspot involved in membrane trafficking and associated with autosomal dominant dRTA. Based on ACMG criteria and its segregation in this family, the variant is classified as likely pathogenic. Variants involving nearby residues (e.g., positions 888, 901, and 905) have been reported in association with dRTA, supporting the functional importance of this region. Functional studies have demonstrated that in autosomal dominant dRTA, mutant kAE1 can form heterodimers with wild-type protein, resulting in mislocalization to apical or mixed membrane domains. This dominant-negative effect impairs bicarbonate transport and contributes to the disease phenotype [11]. SLC4A1-related dRTA exhibits marked phenotypic heterogeneity. In this family, the mother carrying the same variant had a mild phenotype without treatment, whereas the proband developed severe skeletal and renal complications. This discrepancy is consistent with incomplete penetrance and variable expressivity, suggesting that environmental factors, treatment adherence, or modifier genes may influence disease severity.

The patient exhibited profound growth retardation (− 6.2 SD), which is uncommon in SLC4A1-related dRTA. Reported cohorts demonstrate a median height SDS of approximately − 1.96, and adequate alkali therapy has been shown to improve growth outcomes [4]. The severity observed in this case is most likely attributable to prolonged untreated metabolic acidosis. Chronic acidemia suppresses growth plate activity and disrupts bone metabolism [12]. It promotes bone demineralization, inhibits osteoblast function, enhances osteoclastic resorption, may induce secondary hyperparathyroidism, leading to calcium mobilization from bone [6]. Previous studies have described PTH elevation in dRTA as usually mild and reversible after alkali therapy [13]. In contrast, the PTH elevation in this patient was moderate, and the concomitant hypophosphatemia may have been partly related to PTH-mediated renal phosphate wasting, thereby further aggravating impaired bone mineralization.

Radiologically, this case demonstrates classic features of advanced osteomalacia and severe bone demineralization, including generalized osteopenia with a honeycomb trabecular pattern in the distal femoral metaphysis, Looser–Milkman pseudofractures, biconcave “fish” vertebrae, and a trefoil pelvic deformity. These findings indicate long-standing and largely irreversible impairment of bone mineralization. Clinically, the patient exhibits marked functional impairment, including difficulty with ambulation and substantially reduced quality of life [14].

Renal complications represent another critical aspect of dRTA. Regardless of genetic background, nephrocalcinosis and nephrolithiasis are commonly observed, often from early in the disease course, even in patients who achieve metabolic control without severe electrolyte disturbances [15]. Long-term studies have shown that, over a follow-up period of up to 40 years, nearly half of patients develop moderate to severe chronic kidney disease (CKD), although progression to end-stage renal disease is relatively rare. This may be related to the higher burden of complications and episodes of acute electrolyte decompensation during childhood. In addition, obstructive nephrolithiasis and its sequelae (including acute pyelonephritis and acute kidney injury) may contribute to progressive renal damage over time [16].

In the present case, recurrent nephrolithiasis requiring repeated lithotripsy, together with urinary tract infections, ureteral stricture, and hydronephrosis, contributed to a mild decline in renal function consistent with CKD stage 2. Although renal function remains relatively preserved, this clinical course underscores the importance of close surveillance and proactive management to prevent further progression of CKD.

From a therapeutic perspective, alkali supplementation remains the cornerstone of dRTA management, aiming to normalize serum bicarbonate levels and prevent long-term complications. Evidence suggests that with adequate alkali therapy and in the absence of urinary tract infection, the development and progression of nephrocalcinosis and nephrolithiasis can be significantly reduced [17]. However, established structural damage, particularly in long-standing disease, is often irreversible. In this patient, citrate therapy achieved only partial metabolic correction, with persistent suboptimal bicarbonate levels. Recurrent urinary tract infections and nephrolithiasis indicate ongoing disease activity and continued risk of renal damage [18]. These findings highlight the need for treatment optimization, including escalation of alkali dosing to achieve target bicarbonate levels (> 22 mmol/L), strict infection control, and urological management aimed at reducing stone burden. Follow-up should also include serial assessment of 25-hydroxyvitamin D, PTH, alkaline phosphatase, calcium, phosphate, urinary calcium and phosphate handling, urinary citrate, renal function, and renal imaging. After transition to adult care, follow-up was conducted mainly in a local urology department for nephrolithiasis; access to clinicians experienced in dRTA was limited, alkali therapy remained insufficient, and bone/mineral monitoring was incomplete. A structured multidisciplinary pathway involving adult nephrology, endocrinology, urology, and orthopedics may have helped prevent prolonged treatment interruption and ongoing complications.

## Conclusion

This case shows that prolonged interruption of alkali therapy in SLC4A1-related dRTA can lead to severe skeletal and renal complications, even in a genotype usually considered relatively mild. The pediatric-to-adult transition was a key vulnerability, during which treatment continuity and metabolic monitoring were interrupted. Lifelong alkali therapy, regular assessment of acid–base and mineral metabolism and coordinated adult multidisciplinary care are essential to reduce irreversible complications. This report is limited by incomplete follow-up data, particularly serial PTH and 25-hydroxyvitamin D measurements, and by the absence of genetic testing in the father and younger brother.

## Take-home message

- Prolonged interruption of alkali therapy in SLC4A1-related dRTA can result in severe, irreversible skeletal and renal damage, even in genotypes typically considered mild.
- The transition from pediatric to adult care is a critical period for treatment discontinuation, stressing the importance of early intervention and sustained therapy.
- Lifelong alkali therapy, regular metabolic monitoring, and multidisciplinary follow-up (nephrology, endocrinology, orthopedics) are crucial for preventing long-term damage and improving patient outcomes.

## Supplementary Information

Below is the link to the electronic supplementary material.

Supplementary Material 1

## Data Availability

All data generated or analyzed during this study are included in this published article.

## Declarations

### CARE guidelines

This case report has been prepared in accordance with the CARE guidelines.
