# Supplementary material for: Severe skeletal and renal complications in SLC4A1-related distal renal tubular acidosis following prolonged treatment interruption: a case report highlighting transition-related vulnerability
Source: BMC Nephrol. 2026 Aug 8;27:651. doi: 10.1186/s12882-026-05213-4 (PMC13737479; doi:10.1186/s12882-026-05213-4)
Supplement: Supplementary file 1 [file 12882_2026_5213_MOESM1_ESM.pdf]

Untreated Hereditary Distal Renal Tubular Acidosis: Severe Skeletal and Renal Complications

Xinying Gao, Yao Yao, Nan Bai, Xuejing Sun

Abstract

Objective: To demonstrate the severe radiological consequences of prolonged treatment interruption in hereditary dRTA and to emphasize the importance of continuous therapy.

Case Presentation: A 31-year-old female with genetically confirmed hereditary dRTA (SLC4A1 mutation) presented with a 13-year treatment hiatus. Clinical findings included severe metabolic acidosis (pH 7.19, bicarbonate 12.7 mEq/L), hypokalemia, hypophosphatemia, and persistently alkaline urine (pH 7.0). Radiological examination revealed pathognomonic features of severe osteomalacia, including Looser-Milkman pseudofractures, biconcave vertebral deformities, a trefoil pelvis configuration, and extensive bilateral medullary nephrocalcinosis.

Results: The patient demonstrated classical complications of untreated dRTA, including progressive skeletal deformities, recurrent nephrolithiasis requiring annual hospitalization, and chronic kidney disease. Genetic analysis confirmed the presence of a heterozygous SLC4A1 variant (c.2704G>T, p. Asp902Tyr). Following treatment reinitiation, biochemical parameters improved but structural deformities remained irreversible.

Conventions: Abbreviations are usually defined at the first use in the abstract. Check whether 'dRTA' should be defined here. （Line 15-31）

Keywords

Distal renal tubular acidosis, nephrocalcinosis, nephrolithiasis, osteomalacia, skeletal complications.

Introduction

Distal renal tubular acidosis (dRTA), also known as type 1 renal tubular acidosis, is a rare inherited disorder characterized by chronic metabolic acidosis with elevated levels of plasma chloride, associated with an inability to acidify urine[1, 2]. The underlying pathophysiology involves the dysfunction of α-intercalated cells in the collecting duct, resulting in persistently alkaline urine (pH >5.5) even during acidemia[3].

Its global prevalence is approximately 1 in 100,000 live births. Hereditary forms result from mutations in three primary genes: SLC4A1 (encoding anion exchanger AE1), ATP6V1B1, and ATP6V0A4 (encoding proton pump subunits)[4]. Clinical presentations range from asymptomatic cases to severe manifestations including growth retardation, rickets or osteomalacia, nephrolithiasis, nephrocalcinosis, and progressive chronic kidney disease[5, 6].

Early diagnosis and lifelong alkali therapy can prevent most complications and ensure a normal life expectancy[7]. However, delayed diagnosis or treatment interruption leads to severe, often irreversible, skeletal deformities and renal complications[8].

This case demonstrates the devastating radiological consequences of a 13-year treatment hiatus in a patient with a genetically confirmed hereditary dRTA. (line 35-50)

Narrative

Case Presentation

A 31-year-old female with a history of childhood-onset dRTA and a 13-year treatment interruption presented with recurrent nephrolithiasis, progressive pain and weakness, and multiple pathological fractures. Her mother had genu valgum and short stature, with a confirmed diagnosis of dRTA showing compensated metabolic acidosis and alkaline urine, indicating incomplete dRTA.

Physical examination revealed short stature, frontal bossing, severe genu valgum, and joint calcification. Laboratory investigations demonstrated severe metabolic acidosis (arterial pH 7.19; bicarbonate 12.7 mEq/L [12.7 mmol/L]; reference range 22-29 mEq/L [22-29 mmol/L]), hypokalemia (3.0 mEq/L [3.0 mmol/L]; reference range [3.5-5.5 mmol/L]), hypophosphatemia (2.3 mg/dL [0.75 mmol/L]; reference range 2.5-5.0 mg/dL [0.8-1.6 mmol/L]), hypocalcemia (8.6 mg/dL [2.15 mmol/L]; reference range 8.8-10.8 mg/dL [2.2-2.7 mmol/L]), secondary hyperaldosteronism (aldosterone >100 ng/dL [>2770 pmol/L]; reference range 4-31 ng/dL [110-860 pmol/L]), and persistently alkaline urine (pH 7.0).

Radiographs demonstrated severe osteomalacia with characteristic features. Figure 1A shows generalized osteopenia with a honeycomb trabecular pattern in the distal femoral metaphysis (stars). Figure 1B shows pathognomonic Looser-Milkman pseudofractures (arrows) appearing as linear radiolucent bands perpendicular to the cortex. Figure 1C demonstrates the biconcave "fish vertebral " deformities and trefoil pelvis configuration with acetabular protrusion. Figure 1D shows extensive bilateral medullary nephrocalcinosis on CT..

Genetic analysis revealed a heterozygous SLC4A1 variant (c.2704G>T, p. Asp902Tyr) inherited from her mother. The patient required annual hospitalization for nephrolithiasis management over the preceding five years. Following the reinitiation of citrate therapy, the bone pain symptoms showed modest improvement, although the structural deformities remained unchanged.(line 52-76)

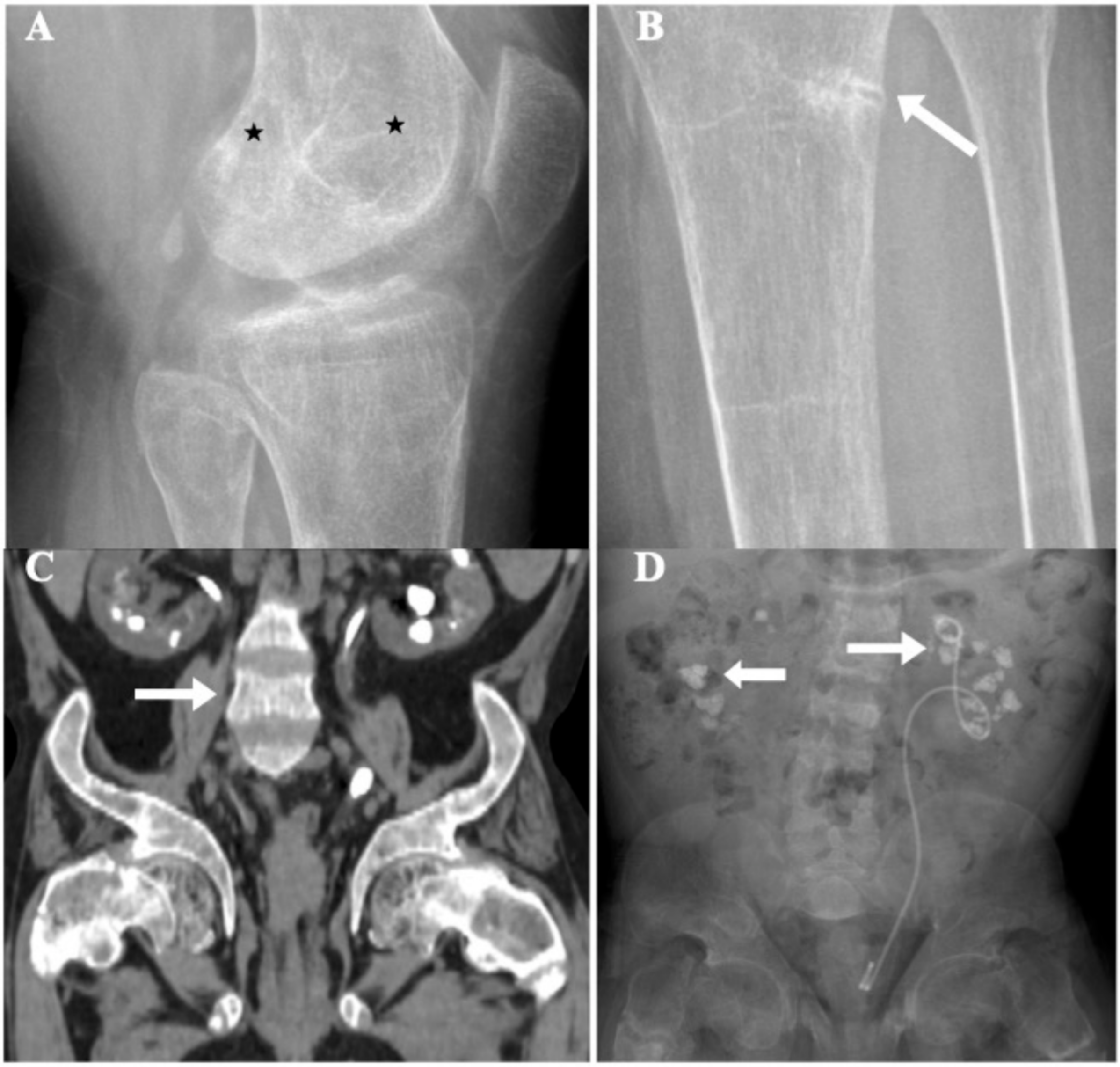

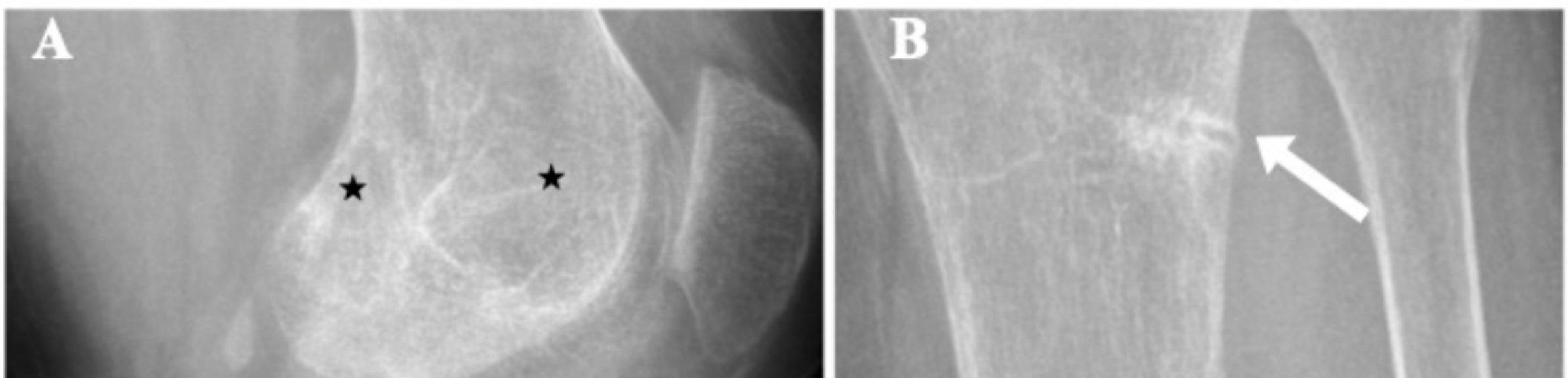

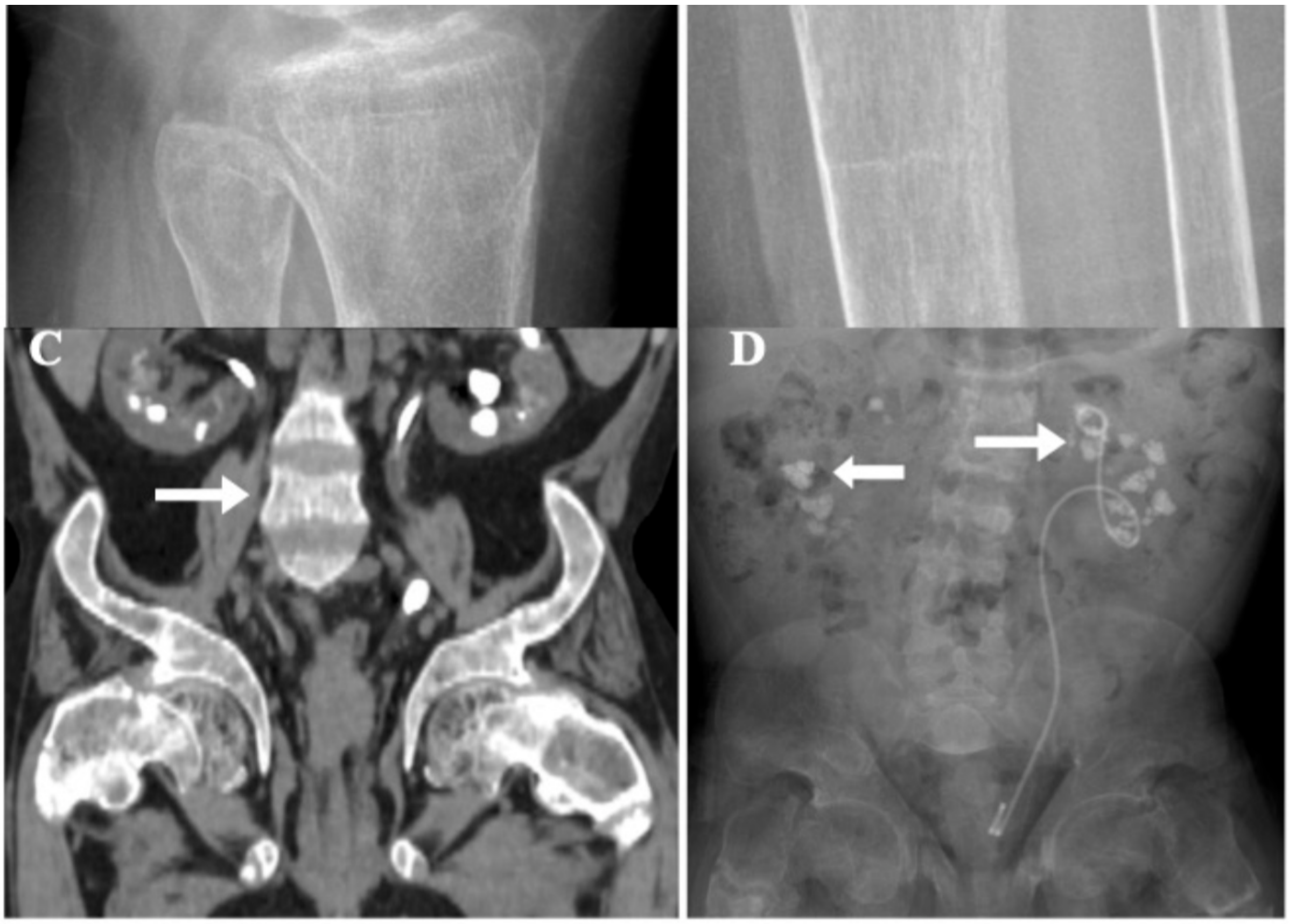

Figure 1: Radiographic Features of Osteomalacia and Nephrocalcinosis

(A) Honeycomb appearance (stars). (B) Looser-Milkman lines (arrow). (C) Fish vertebrae-like changes (arrow) and Trefoil Pelvis. (D) Extensive renal medullary calcification (arrows).

(line178-181)

Timeline

2012-06-05

New Event

The patient exhibited short stature in childhood. Given her mother's diagnosis of dRTA (characterized by short stature and skeletal deformities), the patient was referred to the endocrinology department, where she was diagnosed with renal tubular acidosis and treated with oral citrate. Following her transition from pediatric to adult care at age 18, she discontinued treatment.

2025-01-17

New Event

Because of a 13-year treatment interruption following the transition from pediatric to adult care, the patient presented with recurrent nephrolithiasis, progressive pain and weakness, and multiple pathological fractures. Physical examination revealed short stature, frontal bossing, severe genu valgum, and joint calcification. Laboratory investigations demonstrated severe metabolic acidosis (arterial pH 7.19; bicarbonate 12.7 mEq/L [12.7 mmol/L]; reference range 22-29 mEq/L [22-29 mmol/L]), hypokalemia (3.0 mEq/L [3.0 mmol/L]; reference range [3.5-5.5 mmol/L]), hypophosphatemia (2.3 mg/dL [0.75 mmol/L]; reference range 2.5-5.0 mg/dL [0.8-1.6 mmol/L]), hypocalcemia (8.6 mg/dL [2.15 mmol/L]; reference range 8.8-10.8 mg/dL [2.2-2.7 mmol/L]), secondary hyperaldosteronism (aldosterone >100 ng/dL [>2770 pmol/L]; reference range 4-31 ng/dL [110-860 pmol/L]), and persistently alkaline urine (pH 7.0). Radiographs demonstrated severe osteomalacia with characteristic features. Figure 1A shows generalized osteopenia with a honeycomb trabecular pattern in the distal femoral metaphysis (stars). Figure 1B shows pathognomonic Looser-Milkman pseudofractures (arrows) appearing as linear radiolucent bands perpendicular to the cortex. Figure 1C demonstrates the biconcave "fish vertebral " deformities and trefoil pelvis configuration with acetabular protrusion. Figure 1D shows extensive bilateral medullary nephrocalcinosis on CT.

Diagnostics

TEST/INVESTIGATION	RESULT	UNIT	NOTES
WES	SLC4A1 variant (c.2704G>T, p. Asp902Tyr)		—
arterial pH 7.19	7.19	7.35 -7.45	—
serum bicarbonate	12.7 mEq/L	22-29 mEq/L	—
urine PH	7.0	<5.5	—

Patient Perspective

Following the reinitiation of citrate therapy, the bone pain symptoms showed modest improvement after

6 months, although the structural deformities remained unchanged.(line 74-76)

Discussion

This case provides exceptional radiological evidence of severe osteomalacia secondary to prolonged untreated dRTA. The pathophysiology involves multiple mechanisms. Chronic metabolic acidosis precipitates severe skeletal consequences through a multifaceted pathophysiological cascade. Bone buffering mechanisms release calcium and phosphate from hydroxyapatite crystals, causing progressive demineralization. Direct acidotoxicity inhibits osteoblast-mediated bone formation while stimulating osteoclastic resorption. Secondary hyperparathyroidism, driven by acidosis-induced hypocalcemia, mobilizes skeletal calcium stores. Concurrent phosphaturia leads to hypophosphatemia, which critically impairs mineralization. Disrupted vitamin D metabolism compromises calcium absorption. Collectively, these mechanisms result in childhood rickets that progress to adult osteomalacia characterized by defective bone matrix mineralization[9].

The characteristic imaging findings reflect the systemic nature of mineralization defects. Looser zones (Figure 1B) represent pathognomonic stress fractures that fail to heal owing to defective mineralization. These linear radiolucent bands occur perpendicular to cortical surfaces at sites of mechanical stress or arterial crossings, commonly affecting the femoral neck, pubic rami, ribs, and clavicles. Their bilateral symmetric distribution reflects the systemic nature of mineralization defects [10]. Biconcave vertebral deformities (Figure 1C) result from intervertebral disc expansion into weakened vertebral endplates due to insufficient mineralization. This "fish vertebra" appearance is characteristic of severe osteomalacia and indicates chronic disease progression[11]. Trefoil pelvis is a characteristic deformity observed as a sequela of severe rickets during childhood. Its pathological basis lies in the profound osteomalacia of the pelvic bones during early development, which renders the skeletal structure incapable of withstanding the axial compressive forces of the trunk and the tensile stresses exerted at the muscle attachment sites. This biomechanical imbalance leads to severe distortion of the pelvic inlet and bilateral medial displacement of the iliac bones, culminating in the typical "trefoil" morphology. Concurrently, the femoral heads are forced medially into the pelvis under a compressive load, inducing acetabular protrusion (Protrusio Acetabuli). This radiological sign signifies established and often irreversible structural degradation. While pharmacological interventions can effectively correct underlying metabolic and biochemical abnormalities, they are unable to reverse pre-existing severe biomechanical collapse and skeletal deformities[12]. Extensive bilateral medullary nephrocalcinosis (Fig. 1D) is a direct consequence of the metabolic milieu in dRTA, including hypercalciuria (from bone demineralization and renal calcium leak), hypocitraturia, and persistently alkaline urine, all of which promote the precipitation of calcium phosphate salts in the renal medulla. Once established, this calcification is typically irreversible and poses the risk of progressive chronic kidney disease.(line78-113)

Conclusion

This case provides compelling radiological evidence of severe irreversible complications resulting from untreated hereditary dRTA. These findings emphasize the importance of early diagnosis, lifelong alkali therapy, and seamless care transitions to prevent devastating skeletal and renal sequelae. Healthcare providers must recognize dRTA as a condition requiring continuous management, with particular attention paid to care coordination during pediatric-to-adult transitions.(line 115-121)

Acknowledgements

We are grateful to the Department of Radiology at Beijing Liang Xiang Hospital for their valuable support in imaging and interpretation and to the Department of Urology for their exemplary care provided to the patient during the follow-up period. (line150-153)

Corresponding author: Yao Yao, yaoyaobch@aliyun.com

Author Information

1. **Xinying Gao** (gaoxinying99@gmail.com)

2. **Yao Yao** (yaoyaobch@aliyun.com) **[Corresponding Author]**

3. **Nan Bai** (983306762@qq.com)

4. **Xuejing Sun** (748061072@qq.com)
